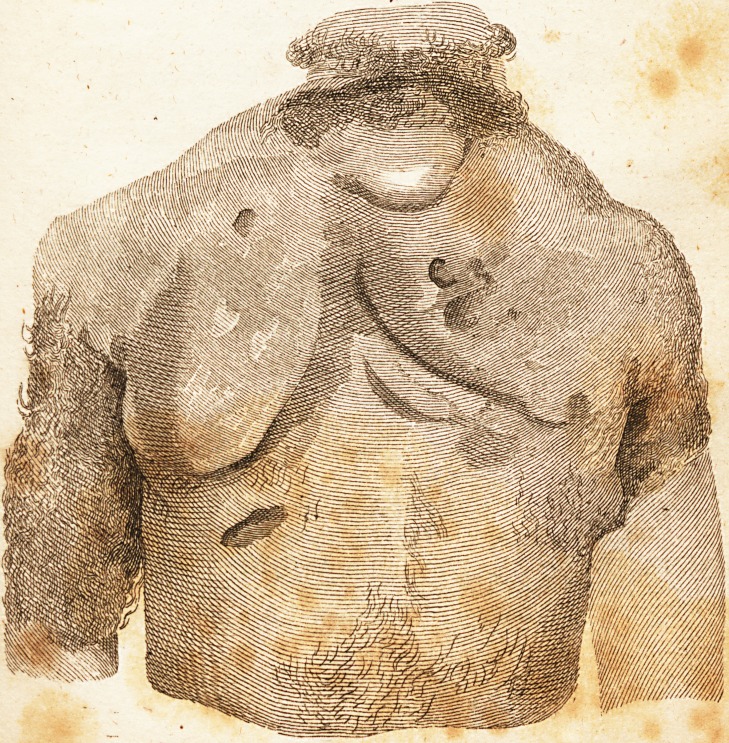# An Inveterate Case of Tinea Capitis

**Published:** 1802-12-01

**Authors:** John Badger

**Affiliations:** Little Scotland Yard, Whitehall


### *An inveterate Case of Tinea Capitis; communicated by Mr.* JOHN BADGER, *of Little Scotland Yard, Whitehall.*

[ With an Engraving. ]

SUSANNAH TAYLOR, aged 60, about four years since, was received into St. Bartholomew's Hospital, with several tumours upon her head the size of a hen's egg, which were removed, and she was discharged soon after perfectly well. However, in a few weeks after this period, she felt a troublesome itching upon her head, which was succeeded by several small pustules; but finding no considerable incovenience from them, she suffered them to remain without any other application than thatof keeping the head clean with soap and warm water, which she

P. 5

ſhe found allayed the itching. In this ſtate, it remained without any appearance of amendment, till about four or five months ago, when ſhe was attacked with a violent fever; but after her recovery, her head becoming gradually worſe, ſhe was again obliged to apply to the Hoſpital for relief, and upon her admiſſion, about two months ſince, the diſeaſe exhibited the extraordinary appearace as ſhewn in the plate. Poultices of linſeed meal were applied for the purpoſe of removing the ſcabs, ſince which ſhe has been uſing the ung. picis. c. ſulphure, but hitherto with very little ſucceſs.

*Aug. 7, 1802.*

## Figures and Tables

**Figure f1:**
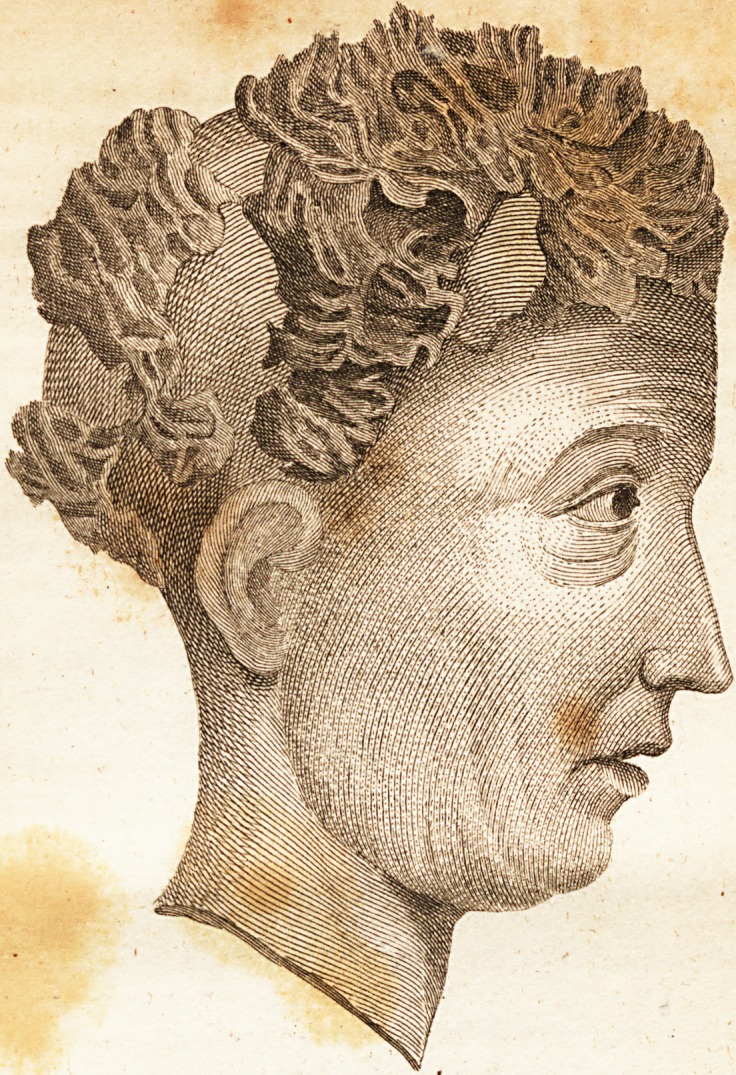


**Figure f2:**